# Population Structure and Growth Dynamics of the Invasive Blue Crab *Callinectes sapidus* in the Loukkos Estuary (Morocco)

**DOI:** 10.3390/biology15040353

**Published:** 2026-02-18

**Authors:** Feirouz Touhami, Hocein Bazairi

**Affiliations:** 1 Laboratory of Biodiversity Ecology and Genome, Faculty of Science, Mohammed V University in Rabat, 4 Avenue Ibn Battouta, B.P. 1014 RP, Rabat 10000, Morocco; hocein.bazairi@fsr.um5.ac.ma; 2 Natural Sciences and Environment Research Hub, University of Gibraltar, Europa Point Campus, Gibraltar GX11 1AA, Gibraltar

**Keywords:** Loukkos estuary, invasive species, *Callinectes sapidus*, biometric characteristics, condition factor, maturity structure

## Abstract

This study provides the first insights into the biology of the blue crab *Callinectes sapidus* in the Loukkos Estuary, based on 461 individuals collected between December 2022 and November 2023. Results indicate a well-structured invasive population. Carapace width ranged from 52 to 201 mm (mean ± SD: 121.7 ± 25.4 mm) and total weight from 12 to 512 g (128.2 ± 76.6 g). Morphometric analyses revealed pronounced sexual dimorphism, with males larger and heavier than females. Size structure shifted seasonally, with smaller crabs dominating spring–summer samples and larger crabs in winter. Biometric relationships were significant and indicated negative allometric growth in both sexes. The sex ratio was strongly male-biased (M/F = 2.72). Condition factor varied with season and sex, peaking in summer and reaching minima in autumn. Female maturity exhibited marked seasonality: immature females prevailed from spring to autumn, whereas mature females occurred mainly in winter. Logistic modeling estimated size at 50% maturity (L_50_) at 126.7 mm carapace width in females. Results suggest that Loukkos Estuary functions primarily as a nursery and growth area for *C. sapidus* and provide essential baseline information for future monitoring and management of this invasive species.

## Simple Summary

The blue crab *Callinectes sapidus* is an invasive species that has recently established populations in several Mediterranean and Atlantic coastal ecosystems. In this study, we examined the biological characteristics of the blue crab population in the Loukkos Estuary using samples collected over one year. Our results showed that this population is well established and structured, with clear differences between males and females and strong seasonal changes in body size, condition, and maturity. Males were generally larger and more abundant than females, and immature females dominated most of the year. The estuary appears to serve mainly as a nursery and growth area, supporting young and developing crabs. These findings provide important baseline information that can support future monitoring and management strategies for this invasive species in a protected estuarine environment.

## 1. Introduction

The Atlantic blue crab *Callinectes sapidus* Rathbun, 1896 is a portunid brachyuran native to the western Atlantic, where it plays a key ecological role in estuarine food webs and supports valuable commercial fisheries [1,2]. Over recent decades, the species has expanded beyond its native range and established self-sustaining populations throughout the Mediterranean Sea and parts of the eastern Atlantic, including the North African Atlantic coast [3,4,5,6,7]. This invasion success has been attributed to a suite of biological traits such as high fecundity, broad environmental tolerance, opportunistic feeding behavior, and efficient larval dispersal, all of which facilitate rapid colonization of transitional coastal environments [2,8,9].

In invaded ecosystems, *C. sapidus* may have ecological and socio-economic impacts. As an opportunistic predator and competitor, it can alter trophic interactions, affect benthic community structure, and potentially influence ecosystem functioning and biodiversity [4,10,11]. At the same time, its increasing abundance has stimulated emerging fisheries and management interest in several Mediterranean and Atlantic regions, highlighting the need for robust biological information to support adaptive management strategies and sustainable exploitation [12,13]. However, the magnitude and nature of these ecological effects appear to depend strongly on local environmental conditions and ecosystem characteristics.

Although population structure, growth patterns, and reproductive traits of *C. sapidus* have been widely documented in both native and invaded areas, increasing evidence indicates substantial spatial variability among populations. Estuarine systems, in particular, are highly dynamic environments characterized by salinity gradients, habitat heterogeneity, seasonal productivity fluctuations, and hydrological connectivity. These factors can influence recruitment success, growth rates, reproductive timing, and spatial distribution of blue crab populations [14,15,16,17]. Understanding how invasive populations respond to these local environmental contexts is therefore essential for assessing establishment success, predicting population trajectories, and evaluating potential ecological impacts.

The Loukkos Estuary, located on the northwestern Atlantic coast of Morocco, represents one of the country’s major estuarine ecosystems and is recognized as a Ramsar wetland of international importance. This productive system supports diverse benthic communities, important fisheries resources, and strong ecological connectivity between freshwater and marine environments. Recent environmental studies conducted in the estuary have documented pronounced spatial variability in salinity, water quality, benthic habitat distribution, and anthropogenic pressures, highlighting its environmental heterogeneity and ecological complexity [18,19]. Such conditions may influence habitat suitability, trophic resources, and life-history traits of estuarine organisms, including invasive species such as *C. sapidus*. Nevertheless, despite the recent expansion of the species along Moroccan coasts [20,21,22], biological information on its populations in North African estuaries remains scarce.

In this context, the present study aims to characterize the biological traits of *C. sapidus* in the Loukkos Estuary while considering the environmental context of this heterogeneous estuarine system. Specifically, the objectives are to (i) describe population structure, including size distribution, sex ratio, and seasonal variability; (ii) analyze biometric relationships and growth patterns; and (iii) examine reproductive traits and condition factor dynamics. By documenting these characteristics and comparing them with those reported from other native and invaded regions, this study provides baseline ecological information necessary for understanding the establishment dynamics of this invasive species and for supporting future monitoring, ecological assessment, and management strategies.

## 2. Materials and Methods

### 2.1. Study Area

**Figure 1:**
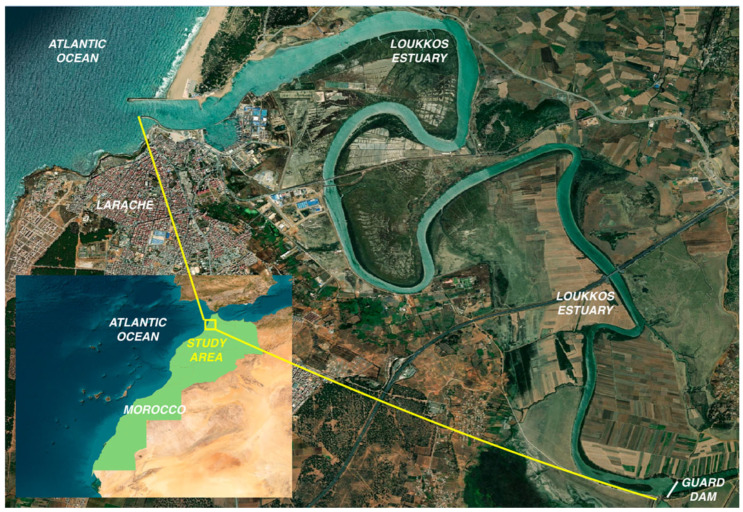
Geographic location of the Loukkos Estuary situated along the northern Atlantic coast of Morocco. The map was produced with QGIS version 3.40.1.

The study was conducted in the Loukkos Estuary, located on the northern Atlantic coast of Morocco near the city of Larache (35°11′ N, 6°09′ W; Figure 1). The estuary forms part of the Loukkos wetland complex, which is characterized by rich biodiversity of national and international importance. This ecological value has led to its designation as a Site of Biological and Ecological Interest and an Important Area for Bird Conservation in Morocco, as well as its inclusion on the Ramsar list of wetlands of international importance since 2005.

The Loukkos Estuary extends over approximately 20 km and is artificially truncated by the Garde Dam (built in 1979), which prevents saltwater intrusion from the Atlantic Ocean and increases freshwater availability for irrigation (Figure 1). Hydrodynamics are driven by the combined influence of freshwater inputs from the Loukkos River and semi-diurnal Atlantic tides. The regional climate is Mediterranean with an oceanic influence, characterized by mild, wet winters and hot, dry summers, with a mean annual air temperature of about 18 °C. Rainfall occurs mainly between November and March. The estuary is dominated by muddy to sandy substrates that support diverse benthic communities and provide essential nursery, feeding, and transit habitats for numerous species [18,19].

### 2.2. Sampling and Morphological Analysis

To characterize the population structure and biological traits of the invasive blue crab in the Loukkos Estuary, a monthly sampling program was conducted over 12 consecutive months, from December 2022 to November 2023. Crabs were collected using baited crab pots operated by a local professional fisherman. The traps were deployed overnight in the middle and lower reaches of the estuary, where the species is most abundant, and retrieved the following day. In total, 461 individuals were analyzed.

For each specimen, a standardized set of morphometric measurements was recorded. Carapace width (CW, mm) was measured as the maximum distance between the tips of the posterior anterolateral spines. Carapace length (CL, mm) was measured along the midline from the frontal margin to the posterior median margin of the carapace. Total weight (TW, g) was obtained using an electronic balance.

Sex was determined based on abdominal morphology: males possess a narrow, elongated T-shaped abdomen, whereas females exhibit a broad, rounded U-shaped abdomen covering the ventral surface. Females were assigned to three categories based on abdominal morphology and reproductive status. Immature females exhibited a narrow, triangular abdomen, whereas mature females displayed a broad, rounded abdomen that fully covered the sternum. Ovigerous females were distinguished by the presence of an external egg mass attached to the pleopods.

### 2.3. Biometric and Reproductive Traits

- Biometric relationships

Relationships between TW and CW, and between TW and CL, were described by the classical allometric models (Equations (1) and (2)):

(1) $$TW=a\;{CW}^{b}$$

(2) $$TW=a\;{CL}^{b}$$

where a is the intercept and b is the allometric coefficient. The equations were fitted using log-transformed data (Equations (3) and (4)):

(3) $$log\left(TW\right)=\log\left(a\right)+b\log\left(CW\right)$$

(4) $$log\left(TW\right)=\log\left(a\right)+b\log\left(CL\right)$$

The value of *b* was used to determine the type of growth: isometric when b = 3, positively allometric when b > 3, and negatively allometric when b < 3.

The relationship between CW and CL was examined using a linear model (Equation (5)):

(5) CL=a+b CW

Isometry in this relationship was tested by comparing the estimated slope to b = 1.

- Condition factor

The Fulton condition factor (K) was used as an index of physiological condition and energy reserves and was calculated as (Equation (6)):

(6) $$K=100\frac{TW}{{CW}^{3}}$$

Higher K values indicate better physiological condition.

- Estimation of size at 50% Maturity (L_50_)

In females, functional maturity was determined from abdominal morphology, with individuals classified as immature or mature according to abdominal shape. The size at 50% maturity (L_50_) was estimated using a binomial generalized linear model (logistic regression) fitted to individual maturity status as a function of carapace width (CW, mm). The probability of maturity was described by the function:

(7) $$P=\frac{1}{1+e^{-r\left(CW-L_{50}\right)}}$$

where P is the probability of maturity at a given CW, r is the slope of the curve, and L_50_ corresponds to the carapace width at which 50% of individuals are mature. The 95% confidence interval for L_50_ was derived from the variance–covariance matrix of the fitted model using the Wald approximation.

### 2.4. Statistical Analyses

Data from the annual survey were grouped into four seasons. Seasons were defined as winter (December–February), spring (March–May), summer (June–August), and autumn (September–November). Seasonal and sexual differences in morphometric parameters were assessed using two-way ANOVA. When significant effects were detected, Tukey’s HSD tests were applied for post hoc comparisons, whereas sex differences within each season were assessed using Welch’s *t*-test. Sex ratio (M/F) was evaluated using chi-square tests against the expected 1:1 proportion. Seasonal differences in female maturity composition were tested using a chi-square (χ^2^) test of independence, followed by pairwise post hoc χ^2^ comparisons to identify significant differences among seasons. All statistical analyses were conducted using R software (v4.5.0).

## 3. Results

### 3.1. Morphometry and Population Structure

**Figure 2:**
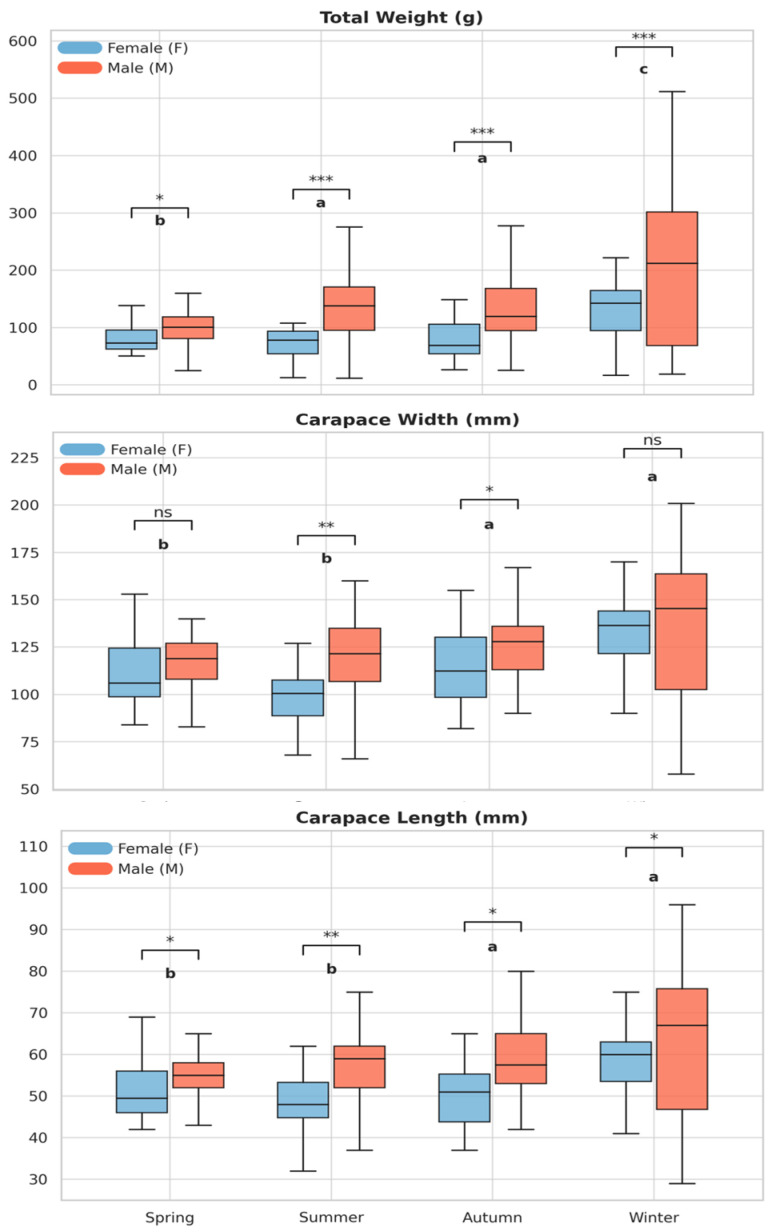
Seasonal variation in total weight, carapace width, and carapace length of *C. sapidus* in females and males. Different letters indicate significant seasonal differences (Tukey’s HSD, *p* < 0.05), and asterisks denote significant sex differences within seasons (Welch’s *t*-test): * *p* < 0.05, ** *p* < 0.01, *** *p* < 0.001; ns = not significant.

A total of 461 individuals were collected (124 females and 337 males). CW ranged from 52 to 201 mm (mean ± SD: 121.7 ± 25.4 mm) in the pooled sample, CL from 25 to 96 mm (56.7 ± 11.4 mm), and TW from 12 to 512 g (128.2 ± 76.6 g) (Table 1). Seasonal differences were observed, with smaller individuals dominating summer samples, whereas winter was characterized by the presence of larger crabs (Table 1; Figure 2).

Two-way ANOVA (Figure 2) revealed significant effects of season and sex on all morphometric traits (*p* < 0.001). Males were significantly larger and heavier than females, and winter individuals were significantly bigger than those collected in other seasons (Tukey HSD, *p* < 0.05). A significant Season × Sex interaction was detected only for total weight (TW; *p* = 0.02).

**Figure 3:**
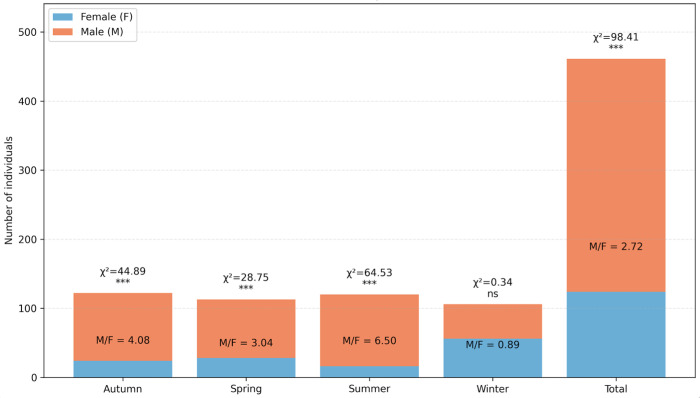
Seasonal and total sex ratio (M/F) of *C. sapidus*. Asterisks denote significance of the chi-square test comparing observed and expected 1:1 ratio: *** *p* < 0.001; ns = not significant.

The global sex ratio was strongly male-biased (M/F = 2.72; χ^2^ = 98.41, *p* < 0.001; Figure 3). Males largely dominated the spring, summer and autumn samples, representing 75–87% of the individuals. In contrast, the winter sample approached parity (M/F = 0.89, *p* = 0.56), with a slight predominance of females.

**Figure 4:**
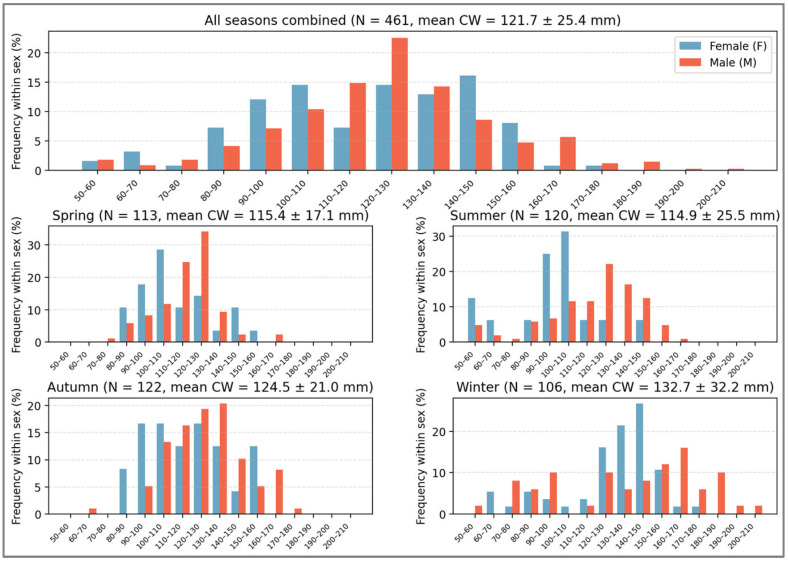
Overall and seasonal size–frequency distribution of carapace width of *C. sapidus*.

The overall CW size-frequency showed unimodal distribution, with a dominant modal class centered on 120–130 mm CW (Figure 4). Males generally occupied broader and larger size classes than females. Marked seasonal shifts in size structure were observed. Spring and summer samples were dominated by small to medium-sized individuals (90–130 mm CW), whereas autumn showed a progressive shift toward larger size classes (110–150 mm CW). Winter exhibited the largest individuals, with a dominant modal class of 130–150 mm CW.

### 3.2. Biometric Relationships

**Figure 5:**
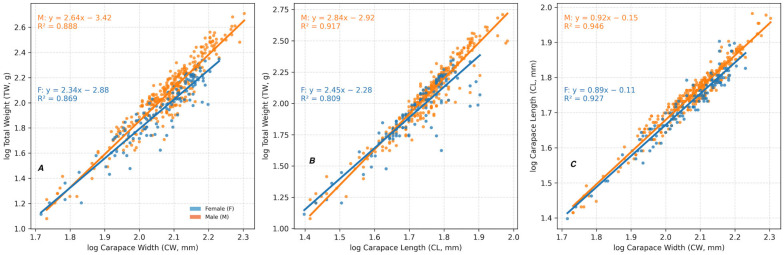
Log–log relationships between (**A**) carapace width and total weight; (**B**) carapace length and total weight; and (**C**) carapace width and carapace length in male and female *C. sapidus*. Regression equations and coefficients of determination (r^2^) are shown on each panel.

All biometric relationships between TW, CW and CL were highly significant for the pooled dataset and for each sex (*p* < 0.001), with strong coefficients of determination (r^2^ = 0.81–0.95; Figure 5). In all cases, growth was negatively allometric, with regression slopes lower than the isometric expectations (b < 3 for TW-CW and TW-CL; b < 1 for CL-CW). Males consistently exhibited slightly higher allometric coefficients and stronger correlations than females.

### 3.3. Condition Factor

**Figure 6:**
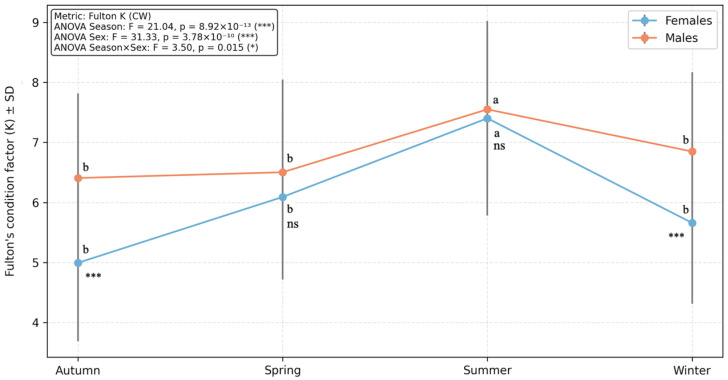
Seasonal and sexual variation in condition factor. Points represent mean values ± standard deviation for each season. Different letters indicate significant seasonal differences within each sex (Tukey’s HSD, *p* < 0.05), and asterisks denote significant differences between sexes within the same season (Welch’s *t*-test): * *p* < 0.05, *** *p* < 0.001; ns = not significant.

The condition factor (K) varied significantly with season and sex (Figure 6). Mean K values peaked in summer and were lowest in autumn. Two-way ANOVA revealed significant effects of season, sex, and their interaction on K (*p* < 0.05). Post hoc tests indicated significantly higher K values in summer compared to spring, autumn, and winter, while sex-related differences were mainly observed in autumn and winter, with males exhibiting higher K values than females. No significant sex differences were detected in spring or summer.

### 3.4. Maturity Structure

**Figure 7:**
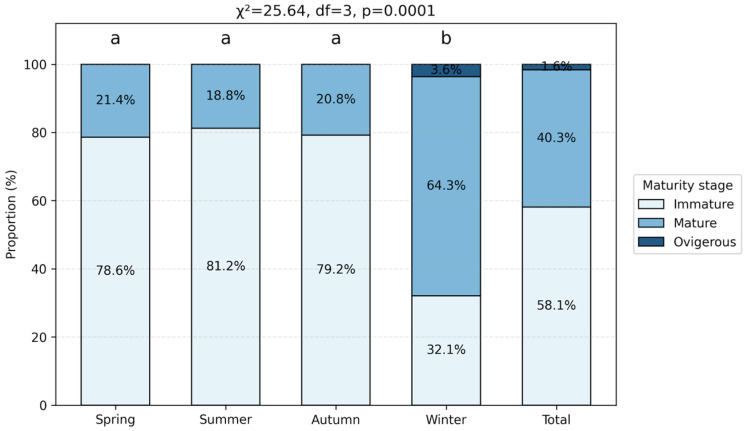
Seasonal maturity composition of female *C. sapidus*. Different letters indicate significant seasonal differences based on chi-square post hoc comparisons (*p* < 0.05). The overall chi-square test result is indicated above the plot.

The seasonal maturity composition of females differed significantly among seasons (χ^2^ = 25.64; *p* < 0.05; Figure 7). Immature females largely dominated the spring, summer and autumn samples, representing more than 78% of individuals in each season. In winter, mature females accounted for 64.3% of the sample, together with a small proportion of ovigerous individuals (3.6%, corresponding to two ovigerous females recorded in February). Post hoc comparisons confirmed that the winter maturity structure differed significantly from that observed during the other seasons (Figure 7).

**Figure 8:**
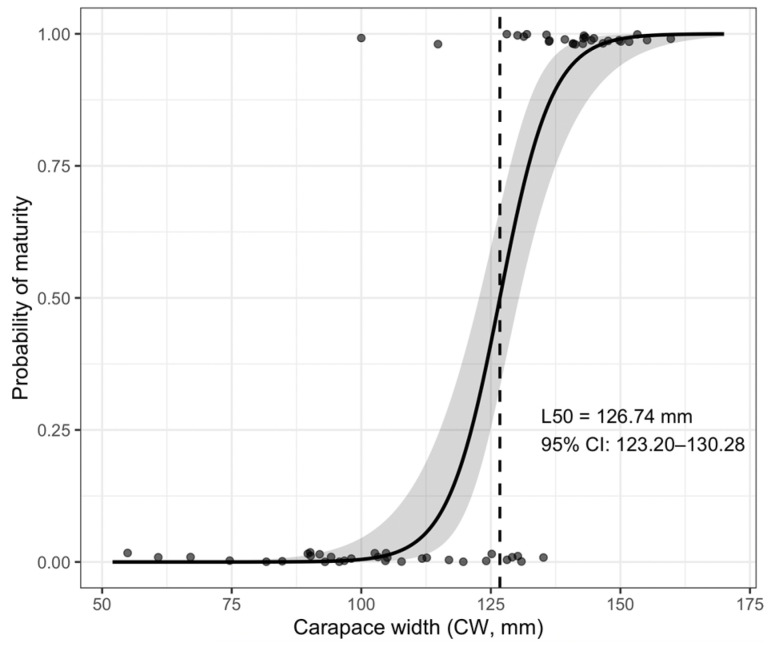
Logistic maturity ogive for female *C. sapidus* estimated using a binomial generalized linear model fitted to individual maturity data. Shaded area indicates the 95% confidence interval.

The estimated size at 50% maturity (L_50_) for female *C. sapidus* collected in the Loukkos Estuary was 126.74 mm CW (95% CI: 123.20–130.28 mm) (Figure 8).

## 4. Discussion

### 4.1. Population Structure and Seasonal Dynamics

This study provides the first insights into the population structure, growth dynamics, and reproductive traits of *Callinectes sapidus* in the Loukkos Estuary. Overall, the population appears well established and demographically structured, as indicated by the broad size range, the seasonal turnover in size classes, and the presence of mature and ovigerous females. Taken together, these features suggest local persistence and the completion of key life-cycle stages within this estuarine system [2,4,9,23,24,25].

The Loukkos Estuary population exhibited clear seasonal shifts in size structure, with smaller individuals predominating during spring and summer and progressively larger crabs dominating in autumn and winter. Comparable seasonal dynamics have been documented in other invaded Mediterranean systems and native Atlantic estuaries, where recruitment pulses followed by rapid growth during warmer months drive pronounced seasonal variation in population size distribution [17,24,26,27].

Temperature is widely recognized as a primary driver of blue crab phenology, influencing metabolic activity, molting frequency, and growth rates [1,28]. Seasonal thermal regimes may therefore partly explain the predominance of smaller individuals during warmer periods and the progressive increase in size toward cooler seasons. In temperate estuaries, larger adults often aggregate in deeper, more stable habitats during colder periods, reducing activity levels and potentially influencing catchability [1,23].

The apparent dominance of medium-to-large size classes in the present study should also be interpreted with caution, as baited pots tend to underrepresent early juvenile stages and emphasize fishery-relevant size classes. This methodological selectivity has been documented in other studies and may partly explain differences between Loukkos and systems where dedicated juvenile sampling was conducted, such as the Trapani saltmarshes in Sicily [24]. Future studies combining multiple sampling approaches would improve resolution of early recruitment dynamics [2].

### 4.2. Sexual Dimorphism and Life-History Strategies

Males were consistently larger and heavier than females, a widespread feature of *C. sapidus* populations across both native and invaded ranges. Sexual dimorphism in portunid crabs is generally interpreted as reflecting sex-specific energy allocation strategies and reproductive roles. Males typically invest more heavily in somatic growth and competitive behaviors that enhance mating success, whereas females incur substantial energetic costs associated with gonadal development and the pubertal molt preceding reproduction [29,30].

These divergent life-history strategies can generate distinct growth trajectories and seasonal body-mass patterns, consistent with the significant Season × Sex interaction observed for total weight in the Loukkos population. Similar patterns have been reported in Mediterranean invaded systems, including Beymelek Lagoon (Turkey), the Evros River estuary (Greece), and the eastern Adriatic coast, suggesting that sexual dimorphism remains relatively stable across contrasting environmental contexts [25,31,32].

### 4.3. Sex Ratio Patterns

The strongly male-biased sex ratio observed in the Loukkos Estuary likely reflects both biological processes and methodological factors. Mature females commonly migrate toward higher-salinity coastal waters for spawning, reducing their representation in estuarine samples, while trap-based sampling tends to preferentially capture larger and more mobile individuals, typically males. Similar biases linked to fishing gear selectivity and sex-specific behavior have been documented in several blue crab studies [1,23,24,33,34].

The rarity of ovigerous females within the estuary further supports the interpretation of spatial reproductive segregation rather than local spawning. In many regions, ovigerous females migrate toward higher-salinity waters where larval survival is enhanced relative to fluctuating estuarine environments [2,35]. Their occasional presence in estuarine habitats likely reflects transient movements or hydrodynamic transport rather than sustained spawning activity [25,33].

Interestingly, the near-parity observed during winter may reflect seasonal behavioral convergence. Reduced activity and aggregation in more stable overwintering habitats may temporarily diminish spatial segregation between sexes, thereby increasing female encounter probability in sampling locations [1,23].

### 4.4. Seasonal Condition Patterns and Energetic Trade-Offs

Seasonal variation in condition factor in the Loukkos population suggests strong environmental modulation of energetic status. Higher condition values during warmer months are consistent with temperature-dependent increases in feeding activity, metabolic performance, and energy storage documented in blue crab populations [28]. Declines later in the year may reflect reduced feeding opportunities, shifts in energy allocation, or increased reproductive investment, particularly in females [29].

Comparable seasonal condition patterns have been reported in Mediterranean invaded populations, where environmental variability and demographic composition strongly influence physiological condition [17,24]. In Loukkos, the emergence of sex-specific differences primarily during autumn and winter suggests that divergent reproductive and maintenance strategies become most apparent when environmental conditions are less favorable.

The biological characteristics observed in the Loukkos Estuary population likely reflect the specific environmental context of this estuarine system, characterized by marked spatial heterogeneity, strong salinity gradients, and high seasonal productivity, particularly during the dry season [18,19]. Estuarine habitats provide abundant benthic prey, structurally complex nursery areas, and transitional physicochemical conditions supporting multiple life stages of *C. sapidus*. Such conditions can enhance juvenile survival, growth, and recruitment while enabling adults to exploit diverse trophic resources. Seasonal recruitment pulses and subsequent growth observed in Loukkos are broadly consistent with patterns described in other productive estuarine systems [36,37]. Nevertheless, direct coupling of environmental and biological datasets will be necessary to clarify causal relationships and better understand how salinity variability, productivity, and hydrological dynamics shape population stability in Atlantic Moroccan estuaries.

### 4.5. Size at Maturity

Size at sexual maturity (L_50_) represents a key indicator for species management, particularly for invasive species, as it provides an estimate of the size at which individuals become reproductively active within a population [38].

The L_50_ estimated for females in the Loukkos Estuary (12.67 cm CW) falls within the range reported for several Mediterranean introduced populations, including Lesina Lagoon, the Trapani saltmarshes, the Evros River estuary, and Beymelek Lagoon [17,24,25,31] (Table 2). This convergence likely reflects broadly comparable estuarine conditions, including intermediate salinity regimes, productive nursery habitats, and connectivity between estuarine and coastal environments that facilitate growth while maintaining access to suitable spawning areas.

In contrast, larger maturity sizes reported from some Corsican lagoons and temperate North American estuaries [39,40,41,42,43] may be associated with environmental conditions favoring prolonged somatic growth prior to reproduction. Cooler thermal regimes generally slow metabolic processes and delay maturation, allowing individuals to attain larger body sizes before reproductive investment [28,44]. Conversely, smaller maturity sizes documented in tropical Brazilian estuaries [45,46] are typically linked to warmer conditions that accelerate growth and reproductive cycles, promoting earlier maturation at smaller sizes. It should also be noted that mature females often migrate toward higher-salinity coastal waters for spawning; consequently, estuarine sampling may underestimate the reproductive segment of the population and potentially bias L_50_ estimates.

**Table 2:** Size at maturity of female *Callinectes sapidus* across native and introduced populations.

Country	Site	Native/ Introduced	Size at Maturity (cm)	Reference
Morocco	Loukkos Estuary	Introduced	12.67	This study
Italy	Lesina Lagoon	Introduced	12.31	[17]
Italy	Trapani saltmarshes	Introduced	12.0	[24]
France	Corsican lagoons	Introduced	13.8–16.7	[43]
Greece	Evros River Estuary	Introduced	12.3–12.4	[25]
Turkey	Beymelek Lagoon	Introduced	11.8	[31]
Brazil	Babitonga Bay	Native	10.2	[45]
Brazil	SE Brazil estuaries	Native	10.33	[46]
USA	St. Johns River	Native	15.0–16.0	[39]
USA	Tampa Bay	Native	13.0	[40]
USA	Texas coast	Native	12.0–13.0	[44]
USA	Chesapeake (Bay tributaries)	Native	14.0	[41]
USA	Chesapeake Bay (lower Bay)	Native	11.8–12.0	[41]
USA	Chesapeake Bay	Native	14.7	[42]

### 4.6. Ecological and Socio-Economic Implications

The establishment of *C. sapidus* in estuarine and lagoon ecosystems has well-documented ecological implications. As an opportunistic omnivore, the species can modify trophic networks through predation on mollusks, crustaceans, and juvenile fish, potentially affecting biodiversity, benthic community structure, and ecosystem functioning [47,48,49,50]. Such trophic flexibility also raises concerns regarding potential competition with native benthic predators and commercially important species, particularly in productive estuarine habitats where resource overlap may occur.

Estuaries frequently function as nursery areas for numerous marine organisms, including fish, crustaceans, and mollusks. The successful establishment of *C. sapidus* in such environments may therefore influence recruitment success of native species through predation on early life stages or competition for shelter and food resources. Although direct ecological impacts have not yet been quantified in the Loukkos Estuary, the ecological characteristics of this system, including high productivity, habitat heterogeneity, and strong estuarine-coastal connectivity could facilitate further population expansion and increase the likelihood of ecological interactions with native communities.

In several Mediterranean regions, particularly in Italy, impacts on bivalve fisheries have generated significant socio-economic concerns, with recent studies reporting measurable effects on fisheries yields and local economic activities [51]. Adaptive management approaches combining ecological monitoring with controlled exploitation have therefore been proposed as pragmatic responses to this invasion, potentially transforming a biological threat into a fishery resource [4,43].

## 5. Conclusions

Population dynamics in the Loukkos Estuary appear to be shaped by both intrinsic life-history traits of *Callinectes sapidus* and the environmental context of this estuarine system, including habitat heterogeneity, seasonal productivity, and physicochemical gradients typical of transitional coastal ecosystems. These conditions likely favor the persistence and potential expansion of the species, although further studies integrating environmental monitoring with population dynamics are needed to clarify the underlying causal mechanisms.

Given its opportunistic feeding behavior and capacity to influence trophic interactions, the continued establishment of *C. sapidus* may have ecological implications for native communities and fisheries resources. Long-term monitoring, expanded spatial sampling, including adjacent coastal habitats, and integrated ecological assessments will therefore be essential for anticipating ecosystem changes and supporting adaptive management strategies for this invasive species.

## Figures and Tables

**Table 1:** Descriptive statistics of morphometric parameters of *C. sapidus* from Loukkos estuary. Values are expressed as mean ± standard deviation (SD), followed by minimum and maximum values. n = number of individuals; F = females; M = males.

Season	Sex	n	Carapace Width (mm)		Carapace Length (mm)		Total Weight (g)	
			Mean ± SD	Min–Max	Mean ± SD	Min–Max	Mean ± SD	Min–Max
Spring	F	28	111.5 ± 18.9	84–153	51.3 ± 7.0	42–69	83.9 ± 31.4	51–159
	M	85	116.6 ± 16.4	73–167	54.9 ± 5.9	37–74	102.6 ± 34.9	25–261
Summer	F	16	96.3 ± 23.7	52–140	46.4 ± 10.5	25–62	69.8 ± 32.4	13–108
	M	104	117.8 ± 24.6	54–160	56.1 ± 10.4	26–75	131.7 ± 59.3	12–290
Autumn	F	24	115.9 ± 22.4	82–155	52.5 ± 11.7	37–78	79.6 ± 34.5	27–149
	M	98	127.1 ± 20.1	60–177	59.1 ± 9.8	29–80	142.3 ± 80.0	26–364
Winter	F	56	128.3 ± 25.6	61–170	57.2 ± 11.3	29–80	125.2 ± 55.4	17–222
	M	50	137.5 ± 38.0	58–201	63.8 ± 18.3	29–96	206.4 ± 129.9	19–512
All Seasons	F	124	118.0 ± 25.6	52–170	53.6 ± 11.0	25–80	100.1 ± 50.0	13–222
	M	337	123.1 ± 25.2	54–201	57.8 ± 11.3	26–96	138.5 ± 82.0	12–512
Total	F + M	461	121.7 ± 25.4	52–201	56.7 ± 11.4	25–96	128.2 ± 76.6	12–512

## Data Availability

The data presented in this study are available on request from the corresponding author.
